# Supplementary figures and images for: Drug use for gastrointestinal symptoms during pregnancy: A French nationwide study 2010–2018
Source: PLoS One. 2021 Jan 22;16(1):e0245854. doi: 10.1371/journal.pone.0245854 (PMC7822332; doi:10.1371/journal.pone.0245854)

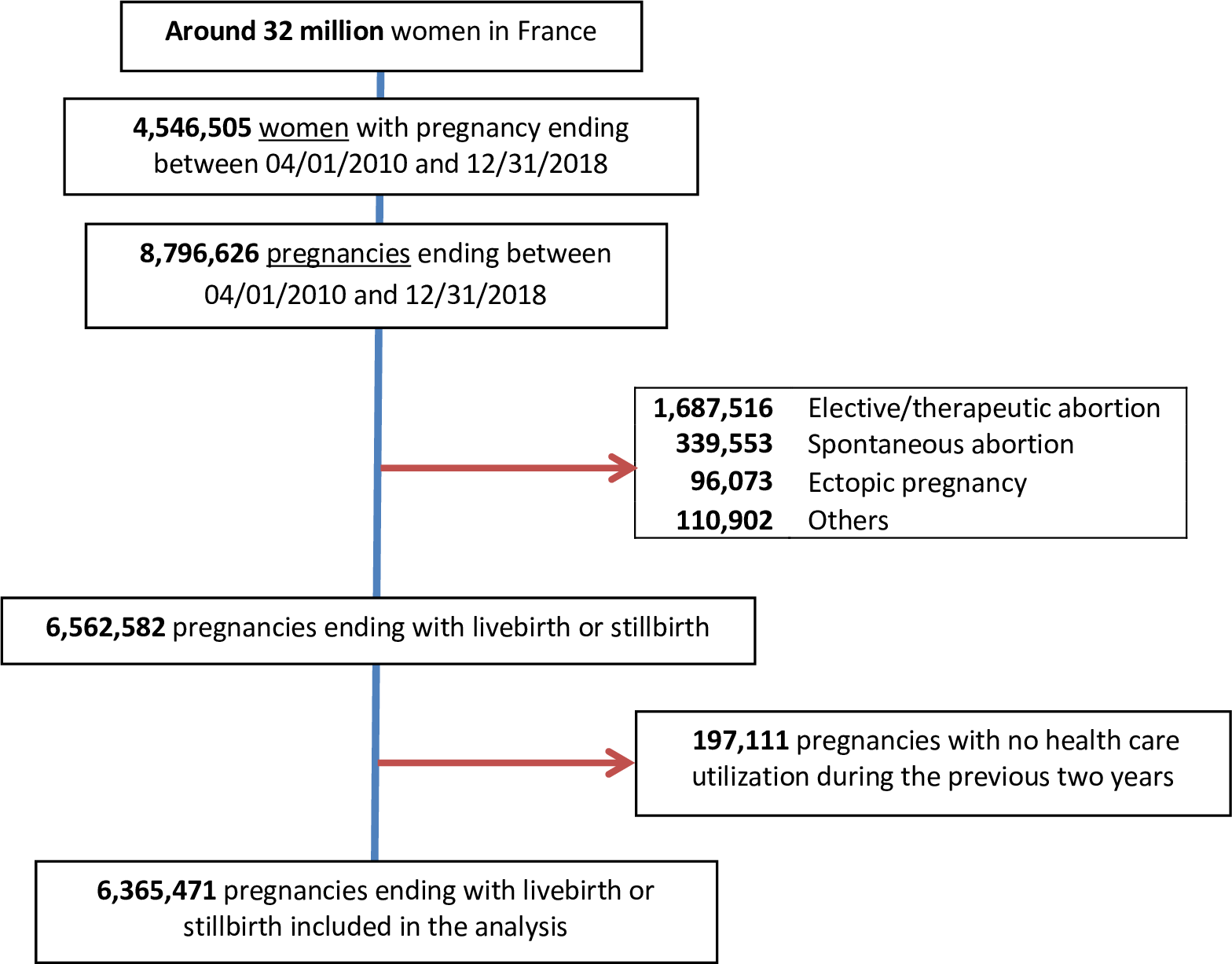

Supplement: S1 Fig — (TIF) [file pone.0245854.s001.tif]

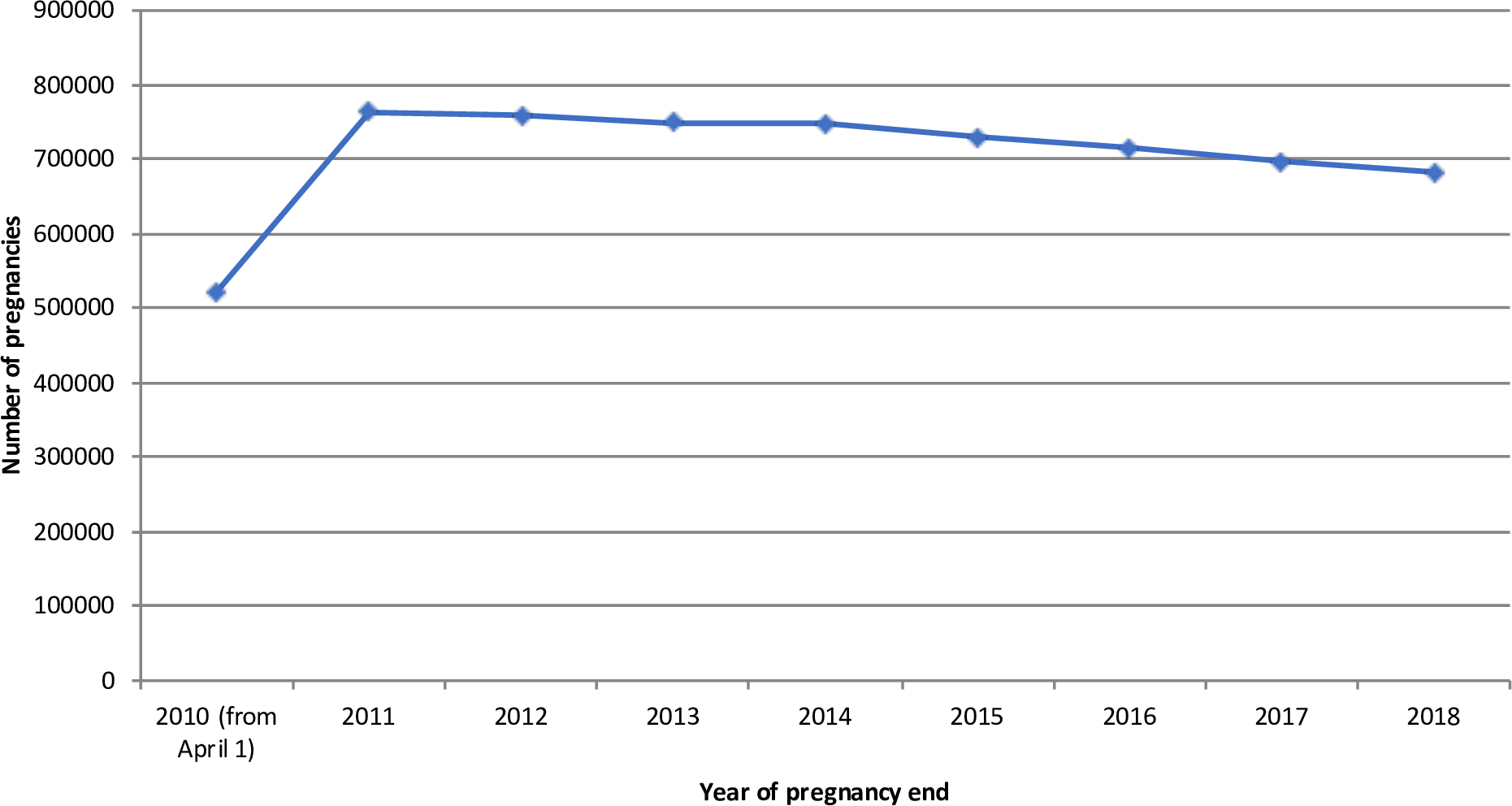

Supplement: S2 Fig — (TIF) [file pone.0245854.s002.tif]

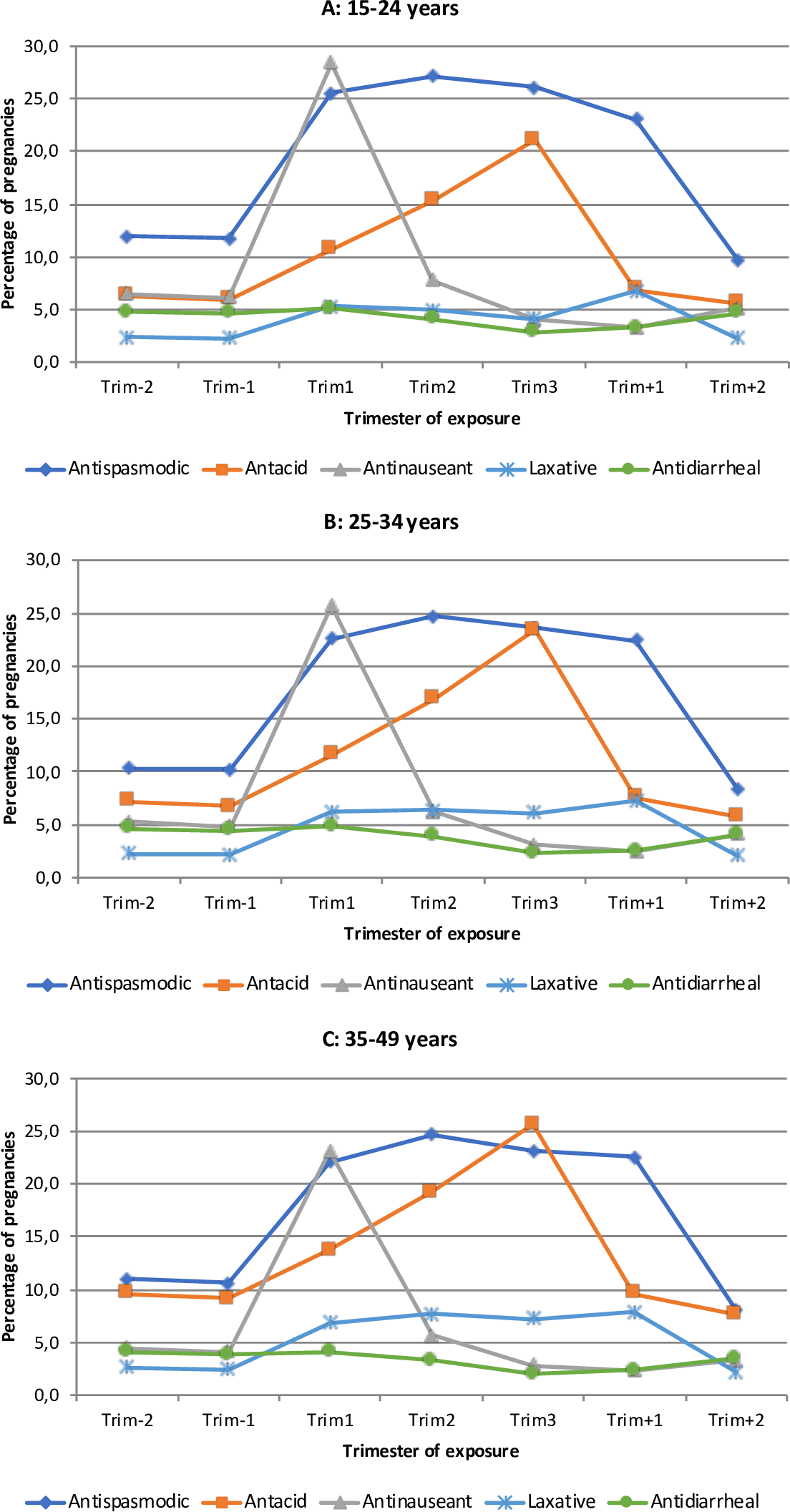

Supplement: S3 Fig — A: 15–24 years; B: 25–34 years; C: 35-49years. Trim: trimester. (TIF) [file pone.0245854.s003.tif]

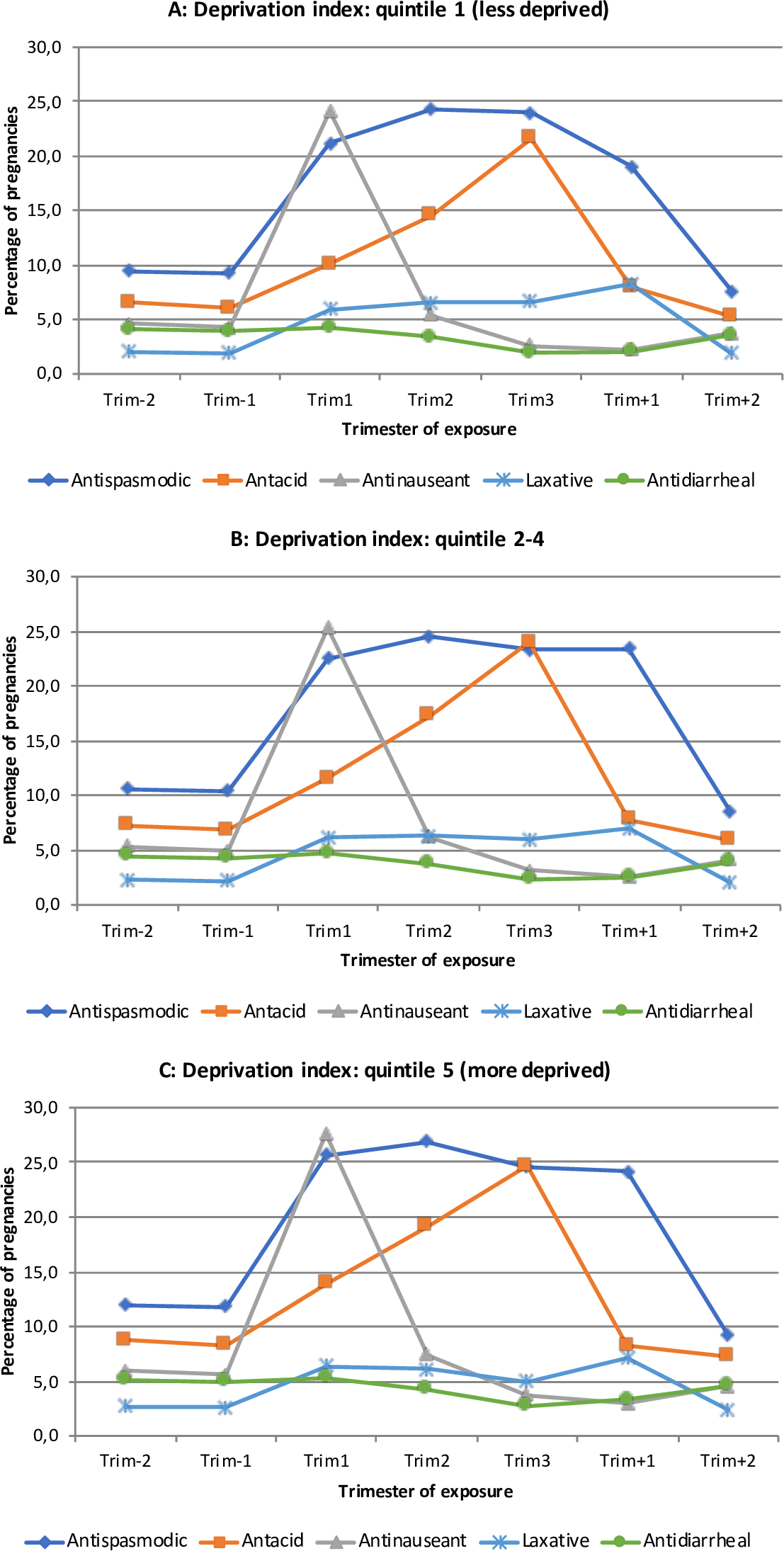

Supplement: S4 Fig — A: quintile 1 (less deprived); B: quintile 2–4; C: quintile 5 (more deprived). The deprivation index expressed in quintiles was developed in France as the first component of a principal component analysis of 4 socioeconomic variables. Trim: trimester. (TIF) [file pone.0245854.s004.tif]

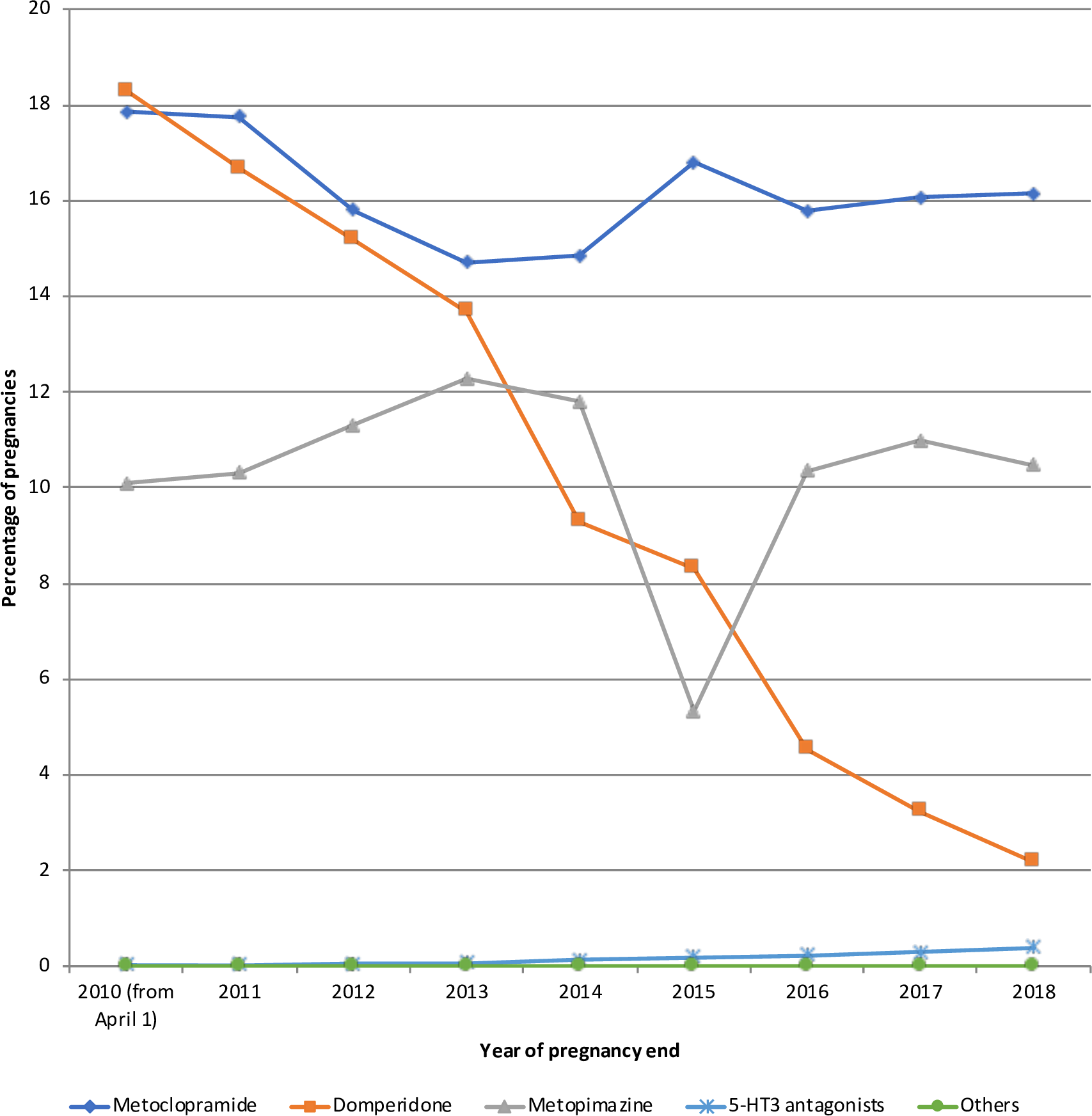

Supplement: S5 Fig — (TIF) [file pone.0245854.s005.tif]

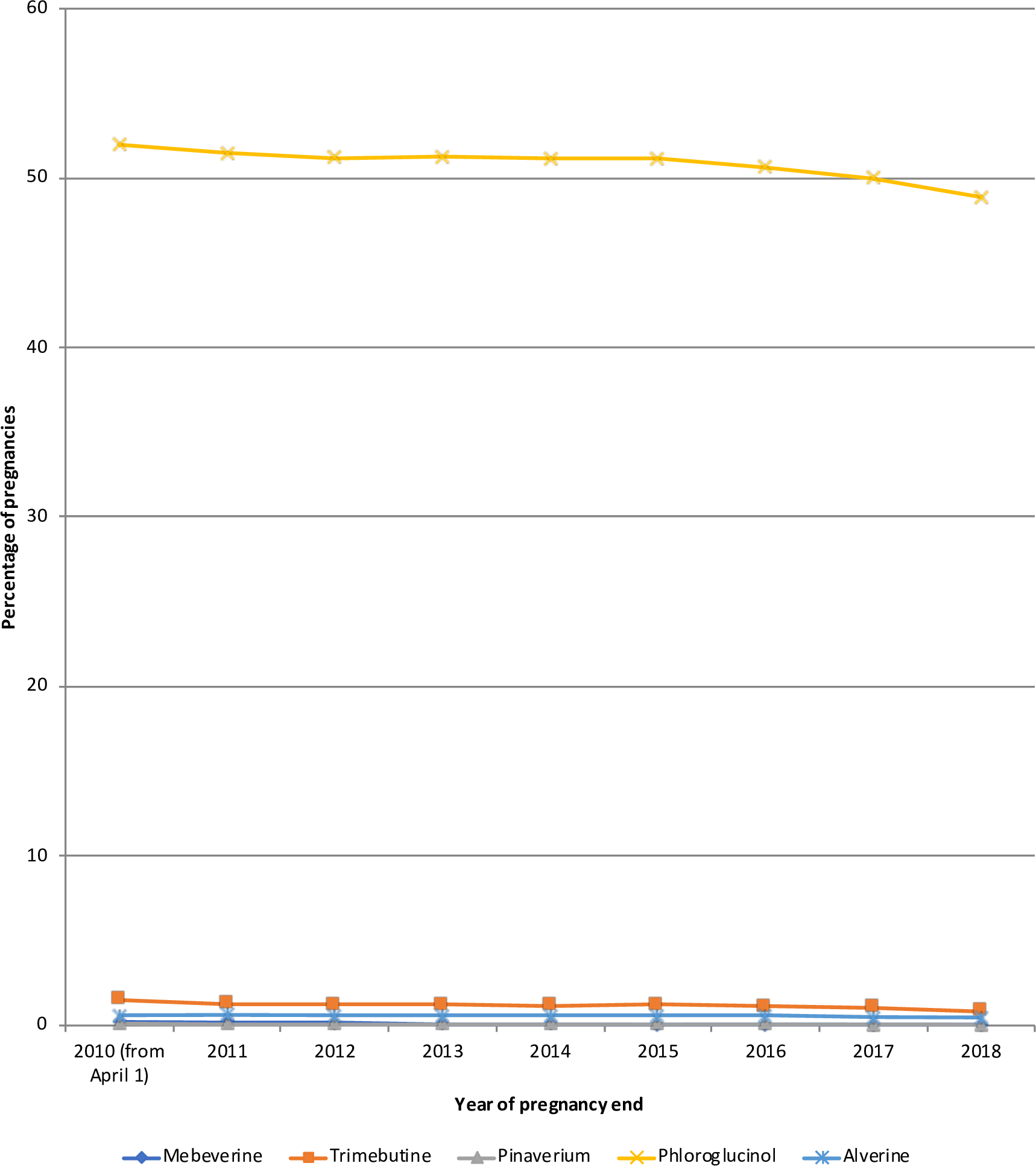

Supplement: S6 Fig — (TIF) [file pone.0245854.s006.tif]

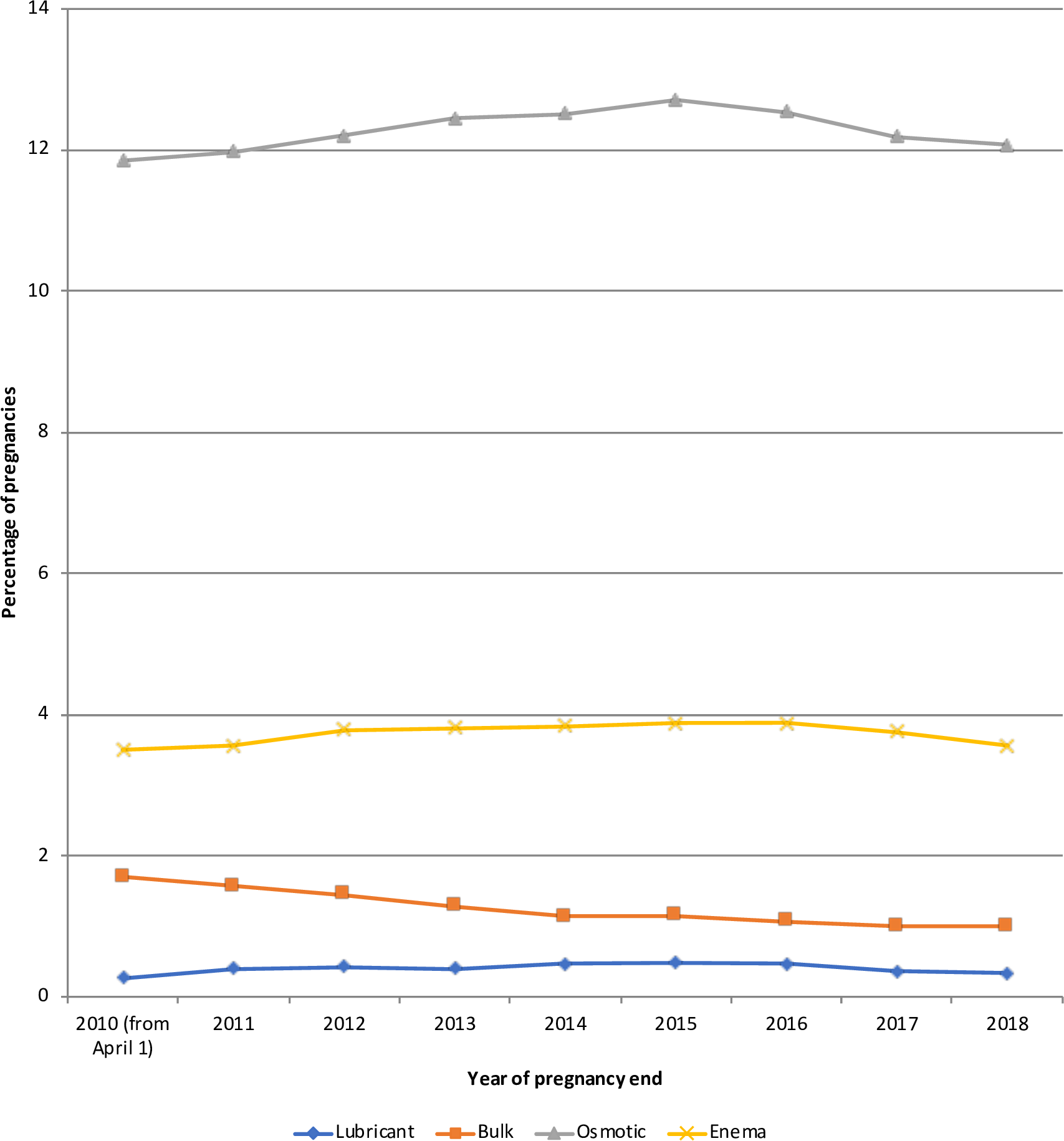

Supplement: S7 Fig — (TIF) [file pone.0245854.s007.tif]

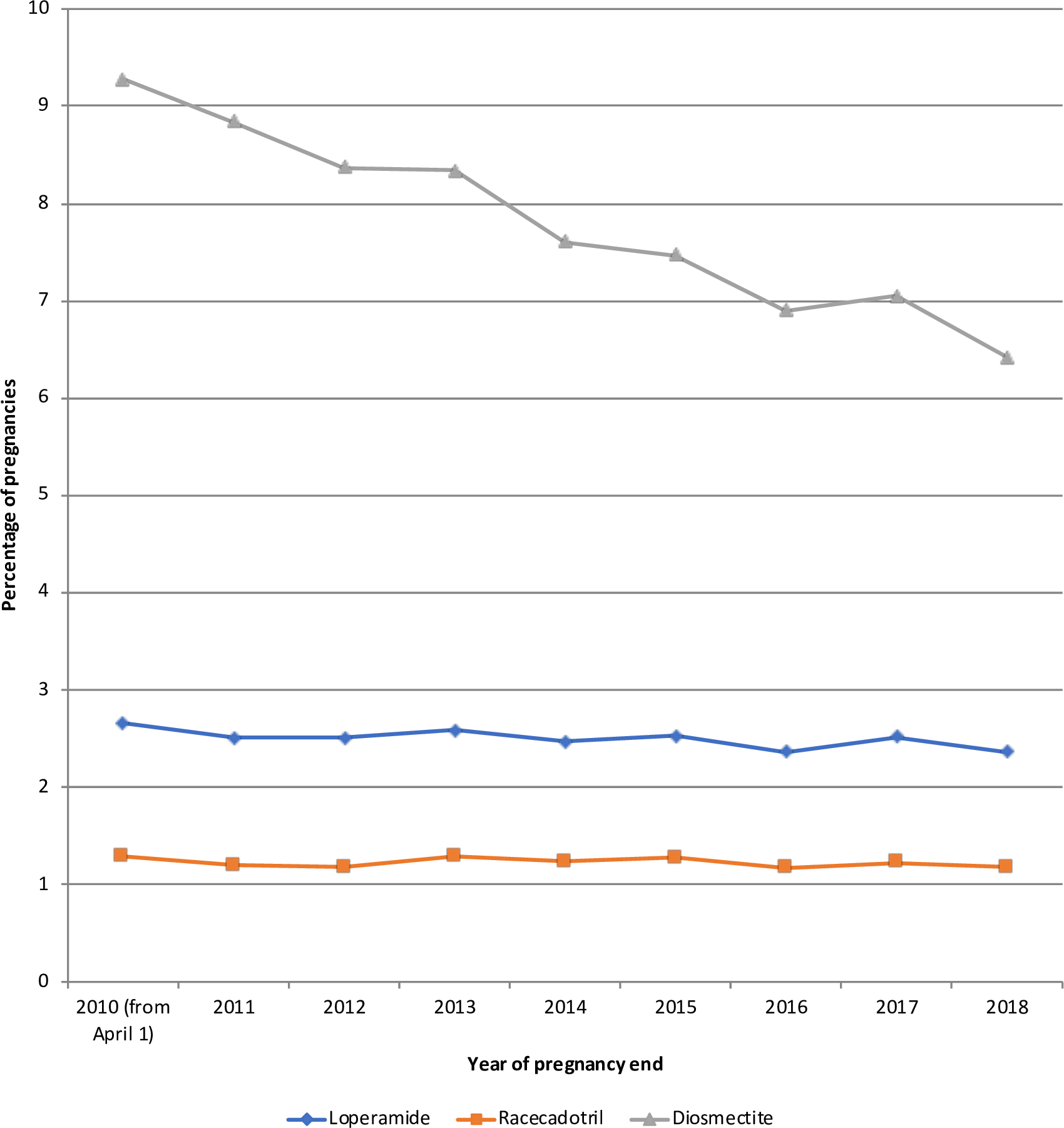

Supplement: S8 Fig — (TIF) [file pone.0245854.s008.tif]
