# Supplementary material for: Drug use for gastrointestinal symptoms during pregnancy: A French nationwide study 2010–2018
Source: PLoS One. 2021 Jan 22;16(1):e0245854. doi: 10.1371/journal.pone.0245854 (PMC7822332; doi:10.1371/journal.pone.0245854)
Supplement: S1 Table — (DOCX) [file pone.0245854.s009.docx]

| **S1 Table. Pregnancy identification algorithms** | |
| --- | --- |
| **Diagnosis** | **Identification algorithm** |
| **Live births** |  |
| Inpatient | [PD/RD/AD: Z37, Z3900 OR PD: O601, O602, O603, O609, O68, O69, O70, O74, O750, O751, O755, O756, O757, O758, O759, O80, O81, O82, O83, O84 OR delivery procedure: CCAM: JQGA002, JQGA003, JQGA004, JQGA005, JQGD001, JQGD002, JQGD003, JQGD004, JQGD005, JQGD007, JQGD008, JQGD010, JQGD012, JQGD013] WITHOUT diagnoses indicative of stillbirth or therapeutic abortion ≥22 weeks of amenorrhea |
| Outpatient | CCAM: JQGD001, JQGD002, JQGD003, JQGD004, JQGD005, JQGD007, JQGD008, JQGD010, JQGD012, JQGD013 by a midwife OR Outpatient procedure 118, 130, 112, 136, 124.8, 151.2 by a midwife |
| **Stillbirths†** | [Before March 2011: AD: Z371, Z373, Z374, Z376, Z377 WITHOUT PD O28, O35, O98, O99 After March 2011 included: AD: Z3710, Z3730, Z3740, Z3760, Z3770 OR PD: O364] WITHOUT [PD O04/O06 OR CCAM JNJD001, JNJD002, JNJP001] |
| **Therapeutic abortions‡** |  |
| ≥22 weeks of amenorrhea | Before March 2011: AD: Z371, Z373, Z374, Z376, Z377 WITHOUT PD O28, O35, O98, O99 After March 2011 included: AD: Z3710, Z3730, Z3740, Z3760, Z3770 OR PD: O04, O06 OR CCAM: JNJD001, JNJD002, JNJP001 |
| <22 weeks of amenorrhea | [PD O04, O05, O06 OR CCAM: JNJD001, JNJD002, JNJP001 WITHOUT PD O00, O01, O02, O03] WITHOUT PD/RD/AD Z640 |
| **Elective abortions§** |  |
| Inpatient | [PD O04, O05, O06 OR CCAM: JNJD001, JNJD002, JNJP001 WITHOUT PD O00, O01, O02, O03] AND PD/RD/AD Z640 |
| Outpatient | CCAM: JNJP001 OR Outpatient procedure 1981, 2411, 2415, 2416, 2419, 2420, 2421, 2422, 2423, 2424, 3329 |
| **Spontaneous abortions¶** | PD: O03 |
| **Ectopic pregnancies** | PD: O00 OR  CCAM: JJFA001, JJFC001, JJJA002, JJJC002, JJLJ001, JJPA001, JJPC001, JQGA001 |
| **Hydatidiform mole or other abnormal products of conception** | PD: O01, O02 |
| *If not otherwise specified, the codes are ICD-10 codes. † Stillbirth: death of a fetus with a gestational age ≥ 22 weeks of amenorrhea. ‡ Therapeutic abortion: termination of pregnancy for maternal health or fetal disease. § Elective abortion: termination of pregnancy at the woman's request for reasons other than maternal health or fetal disease, possible until 14 weeks of amenorrhea in France. ¶ Spontaneous abortion: death of a fetus with gestational age < 22 weeks of amenorrhea. ICD-10: International Classification of Disease, 10th revision. CCAM: French medical classification of clinical procedures. PD: Principal diagnoses. RD: Related diagnoses. AD: Associated diagnoses. PD/RD/AD are hospital discharge diagnoses and PD/RD correspond to the diseases justifying hospitalization.* | |
