# Supplementary material for: Drug use for gastrointestinal symptoms during pregnancy: A French nationwide study 2010–2018
Source: PLoS One. 2021 Jan 22;16(1):e0245854. doi: 10.1371/journal.pone.0245854 (PMC7822332; doi:10.1371/journal.pone.0245854)
Supplement: S2 Table — (DOCX) [file pone.0245854.s010.docx]

| **S2 Table. Drugs** | | |
| --- | --- | --- |
| **Therapeutic class** | **International non-proprietary name** | **Anatomical Therapeutic Chemical (ATC) Classification** |
| **Antacids** |  |  |
| Locally-acting | Aluminium hydroxide, aluminium phosphate, magnesium hydroxide, magnesium aluminosilicate, sucralfate, alginate | A02A, A02BX |
| Histamine 2 blocker | Cimetidine, ranitidine, famotidine, nizatidine, roxatidine | A02BA |
| Proton pump inhibitor | Omeprazole, pantoprazole, lansoprazole, rabeprazole, esomeprazole | A02BC |
| **Antispasmodics** |  |  |
| Mebeverine | Mebeverine | A03AA04 |
| Trimebutine | Trimebutine | A03AA05 |
| Pinaverium | Pinaverium | A03AX04 |
| Phloroglucinol | Phloroglucinol | A03AX12, A03ED |
| Alverine | Alverine | A03AX58 |
| Others | Tiemonium iodide, simeticone, dihexyverine | A02AX, A03AA08, A03AB17, A03DC, A07BA51, A07BA51 |
| **Antinauseants** |  |  |
| Metoclopramide | Metoclopramide | A03FA01 |
| Domperidone | Domperidone | A03FA03 |
| Metopimazine | Metopimazine | A04AD05 |
| 5-HT3 antagonists | Ondansetron, granisetron, tropisetron, dolasetron, palonosetron | A04AA |
| Others | Aprepitant, rolapitant, alizapride | A04AD12, A04AD14, A03FA05 |
| **Laxatives** |  |  |
| Lubricant | Liquid paraffin | A06AA |
| Bulk | Ispaghul, sterculia | A06AC |
| Osmotic | Lactulose, lactitol, pentaerithrityl, macrogol, sorbitol | A06AD |
| Enema | Hydrogenophosphate, tartrate acid, bicarbonate | A06AG, A06AX |
| Others | Methylnaltrexone bromide, naloxegol | A06AH |
| **Antidiarrheals** |  |  |
| Loperamide | Loperamide | A07DA |
| Racecadotril | Racecadotril | A07XA |
| Diosmectite | Diosmectite | A02X |
| **Ursodeoxycholic acid** | Ursodeoxycholic acid | A05AA |
| **Other drugs for functional gastrointestinal disorders** | Dimeticone, povidone, eseridine, beidellitic montmorillonite | A02AX, A02X, A03AX13, A07BA51 |
| **All gastrointestinal drugs** | All drugs above |  |
| **ATC A02-A09** |  | A02-A09 |
