# Supplementary material for: Drug use for gastrointestinal symptoms during pregnancy: A French nationwide study 2010–2018
Source: PLoS One. 2021 Jan 22;16(1):e0245854. doi: 10.1371/journal.pone.0245854 (PMC7822332; doi:10.1371/journal.pone.0245854)
Supplement: S3 Table — (DOCX) [file pone.0245854.s011.docx]

| **S3 Table. Hospitalization for gastrointestinal diseases** | |
| --- | --- |
| **Disease** | **International Classification of Disease, 10th revision:**  **principal diagnosis** |
| Nausea/Vomiting | O21, R11 |
| Cholestasis | K710, K831, O266 |
| Proctological disease (fissure, fistula, abscess, hemorrhoids) | I84, K60, K61, K64, L05, O224, O872 |
| Appendicitis | K35, K36, K37 |
| Biliary tract disease | K80, K81, K830, K851 |
