# Supplementary material for: Drug use for gastrointestinal symptoms during pregnancy: A French nationwide study 2010–2018
Source: PLoS One. 2021 Jan 22;16(1):e0245854. doi: 10.1371/journal.pone.0245854 (PMC7822332; doi:10.1371/journal.pone.0245854)
Supplement: S4 Table — (DOCX) [file pone.0245854.s012.docx]

| **S4 Table. Drug exposure (thousands): sensitivity analysis excluding pregnancies lasting less than 37 weeks** | | | | | | | | |
| --- | --- | --- | --- | --- | --- | --- | --- | --- |
|  | **Number of pregnancies, thousands (%)** | | | | | | | |
|  | **Trim-2** | **Trim-1** | **Trim1** | **Trim2** | **Trim3** | **Trim123** | **Trim+1** | **Trim+2** |
| **Number of pregnancies** | 5,612 | 5,612 | 5,612 | 5,612 | 5,612 | 5,612 | 5,612 | 5,612 |
| **Antacids** | 413 (7.4) | 387 (6.9) | 657 (11.7) | 944 (16.8) | 1,329 (23.7) | 2,084 (37.1) | 434 (7.7) | 334 (6.0) |
| Locally-acting | 106 (1.9) | 96 (1.7) | 416 (7.4) | 624 (11.1) | 769 (13.7) | 1,443 (25.7) | 98 (1.8) | 89 (1.6) |
| Histamine 2 blocker | 10 (0.2) | 9 (0.2) | 19 (0.3) | 41 (0.7) | 74 (1.3) | 111 (2.0) | 7 (0.1) | 6 (0.1) |
| Proton pump inhibitor | 350 (6.2) | 332 (5.9) | 342 (6.1) | 427 (7.6) | 713 (12.7) | 1,109 (19.8) | 366 (6.5) | 279 (5.0) |
| **Antispasmodics** | 593 (10.6) | 584 (10.4) | 1,275 (22.7) | 1,386 (24.7) | 1,334 (23.8) | 2,864 (51.0) | 1,256 (22.4) | 477 (8.5) |
| Mebeverine | 10 (0.2) | 9 (0.2) | 4 (0.1) | 1 (0.0) | 0 (0.0) | 6 (0.1) | 3 (0.1) | 5 (0.1) |
| Trimebutine | 78 (1.4) | 77 (1.4) | 42 (0.7) | 19 (0.3) | 9 (0.2) | 67 (1.2) | 68 (1.2) | 59 (1.0) |
| Pinaverium | 12 (0.2) | 11 (0.2) | 4 (0.1) | 1 (0.0) | 0 (0.0) | 5 (0.1) | 4 (0.1) | 6 (0.1) |
| Phloroglucinol | 490 (8.7) | 484 (8.6) | 1,236 (22.0) | 1,373 (24.5) | 1,328 (23.7) | 2,835 (50.5) | 1,198 (21.4) | 404 (7.2) |
| Alverine | 53 (0.9) | 53 (1.0) | 24 (0.4) | 6 (0.1) | 2 (0.0) | 32 (0.6) | 21 (0.4) | 35 (0.6) |
| Others | 10 (0.2) | 10 (0.2) | 7 (0.1) | 3 (0.1) | 1 (0.0) | 11 (0.2) | 4 (0.1) | 6 (0.1) |
| **Antinauseants** | 297 (5.3) | 276 (4.9) | 1,442 (25.7) | 357 (6.4) | 182 (3.2) | 1,737 (30.9) | 140 (2.5) | 231 (4.1) |
| Metoclopramide | 46 (0.8) | 39 (0.7) | 748 (13.3) | 157 (2.8) | 79 (1.4) | 904 (16.1) | 20 (0.4) | 32 (0.6) |
| Domperidone | 150 (2.7) | 138 (2.5) | 477 (8.5) | 106 (1.9) | 53 (1.0) | 592 (10.6) | 73 (1.3) | 102 (1.8) |
| Metopimazine | 113 (2.0) | 109 (1.9) | 458 (8.2) | 109 (1.9) | 55 (1.0) | 580 (10.3) | 50 (0.9) | 104 (1.9) |
| 5-HT3 antagonists | 0 (0.0) | 0 (0.0) | 6 (0.1) | 2 (0.0) | 1 (0.0) | 7 (0.1) | 0 (0.0) | 1 (0.0) |
| Others | 0 (0.0) | 0 (0.0) | 0 (0.0) | 0 (0.0) | 0 (0.0) | 0 (0.0) | 0 (0.0) | 1 (0.0) |
| **Laxatives** | 131 (2.3) | 122 (2.2) | 343 (6.1) | 351 (6.3) | 337 (6.0) | 859 (15.3) | 416 (7.4) | 118 (2.1) |
| Lubricant | 8 (0.1) | 8 (0.1) | 12 (0.2) | 8 (0.1) | 5 (0.1) | 23 (0.4) | 17 (0.3) | 8 (0.1) |
| Bulk | 17 (0.3) | 16 (0.3) | 33 (0.6) | 29 (0.5) | 19 (0.3) | 71 (1.3) | 26 (0.5) | 15 (0.3) |
| Osmotic | 100 (1.8) | 93 (1.7) | 292 (5.2) | 300 (5.3) | 222 (4.0) | 689 (12.3) | 338 (6.0) | 92 (1.6) |
| Enema | 27 (0.5) | 25 (0.4) | 55 (1.0) | 54 (1.0) | 121 (2.2) | 213 (3.8) | 88 (1.6) | 21 (0.4) |
| Others | 0 (0.0) | 0 (0.0) | 0 (0.0) | 0 (0.0) | 0 (0.0) | 0 (0.0) | 0 (0.0) | 0 (0.0) |
| **Antidiarrheals** | 255 (4.6) | 245 (4.4) | 267 (4.8) | 215 (3.8) | 135 (2.4) | 580 (10.3) | 146 (2.6) | 228 (4.1) |
| Loperamide | 91 (1.6) | 86 (1.5) | 67 (1.2) | 49 (0.9) | 31 (0.5) | 141 (2.5) | 43 (0.8) | 71 (1.3) |
| Racecadotril | 103 (1.8) | 99 (1.8) | 42 (0.7) | 19 (0.3) | 10 (0.2) | 69 (1.2) | 50 (0.9) | 95 (1.7) |
| Diosmectite | 112 (2.0) | 107 (1.9) | 191 (3.4) | 168 (3.0) | 108 (1.9) | 445 (7.9) | 79 (1.4) | 106 (1.9) |
| **Ursodeoxycholic acid** | 2 (0.0) | 2 (0.0) | 1 (0.0) | 2 (0.0) | 16 (0.3) | 18 (0.3) | 2 (0.0) | 2 (0.0) |
| **All gastrointestinal drugs** | 1,105 (19.7) | 1,058 (18.9) | 2,433 (43.4) | 2,263 (40.3) | 2,449 (43.6) | 4,146 (73.9) | 1,861 (33.2) | 933 (16.6) |
| **ATC A02-A09** | 1,116 (19.9) | 1,069 (19.1) | 2,438 (43.4) | 2,267 (40.4) | 2,454 (43.7) | 4,149 (73.9) | 1,874 (33.4) | 944 (16.8) |
| *Medication dispensing before, during and after pregnancy was described for pregnancies ending between April 2010 and June 2018: crude numbers and percentages of exposed pregnancies by trimester. As other drugs for functional gastrointestinal disorders represented <0.1% for each trimester, they were not reported. ATC: Anatomical Therapeutic Chemical.* | | | | | | | | |
